# Challenges and opportunities for silicosis prevention and control: need for a national health program on silicosis in India

**DOI:** 10.1186/s12995-023-00379-1

**Published:** 2023-07-11

**Authors:** Mihir P. Rupani

**Affiliations:** Clinical Epidemiology (Division of Health Sciences), ICMR - National Institute of Occupational Health (NIOH), Indian Council of Medical Research, Meghaninagar, Ahmedabad city, 380016 Gujarat India

**Keywords:** Silica dust control, Awareness, Statutory provisions, Surveillance, Law enforcement, India

## Abstract

**Background:**

Silicosis has been one of the most serious occupational public health problems worldwide for many decades. The global burden of silicosis is largely unknown, although it is thought to be more prevalent in low and medium-income countries. Individual studies among workers exposed to silica dust in various industries, however, reveal a high prevalence of silicosis in India. This paper is an updated review of the novel challenges and opportunities for silicosis prevention and control in India.

**Main body:**

The unregulated informal sector employs workers on contractual appointment thereby insulating the employers from legislative provisions. Due to a lack of awareness of the serious health risks and low-income levels, symptomatic workers tend to disregard the symptoms and continue working in dusty environments. To prevent any future dust exposure, the workers must be moved to an alternative job in the same factory where they will not be exposed to silica dust. Government regulatory bodies, on the other hand, must guarantee that factory owners relocate workers to another vocation as soon as they exhibit signs of silicosis. Technological advances such as artificial intelligence and machine learning might assist industries in implementing effective and cost-saving dust control measures. A surveillance system needs to be established for the early detection and tracking of all patients with silicosis. A pneumoconiosis elimination program encompassing health promotion, personal protection, diagnostic criteria, preventive measures, symptomatic management, prevention of silica dust exposure, treatment, and rehabilitation is felt important for wider adoption.

**Conclusion:**

Silica dust exposure and its consequences are fully preventable, with the benefits of prevention considerably outweighing the benefits of treating patients with silicosis. A comprehensive national health program on silicosis within the public health system would strengthen surveillance, notification, and management of workers exposed to silica dust in India.

## Background

Silicosis is an irreversible and fatal pneumoconiosis affecting millions of workers worldwide who are exposed to silica dust [1]. Mining, tunneling, quarrying, sculpturing, foundries, and manufacturing of building construction materials are some of the key industries in which workers are exposed to silica dust [2]. Although silicosis is considered to be more prevalent in low and middle-income countries, evidence on its global burden is limited, due mostly to underreporting and low detection due to a lack of surveillance [1, 3–6]. In 2019, an estimated 2.65 million cases of silicosis were reported worldwide [7]. Globally, in 2019, over 12.9 thousand deaths and 0.65 million disability-adjusted life years (DALYs) were attributed to silicosis, accounting for three-fourths of all pneumoconiosis DALYs [1, 7]. Many workers develop silicosis after 5–10 years of being exposed to silica dust and die within 15 years, undermining the seriousness of the disease [1]. The permissible exposure limit (PEL) for silica dust in Indian mines is set at 0.15 mg/m3, calculated as an 8-hour time-weighted average [8]. In contrast, the U.S. Occupational Safety and Health Administration (OSHA) prescribes a PEL of 0.05 mg/m^3^ and an action level of 0.025 mg/m^3^ [9]. Similarly, the American Conference of Government Industrial Hygienists (ACGIH) sets a threshold limit value of 0.025 mg/m^3^ [10]. These variations indicate that the prescribed limits for crystalline silica dust in India are significantly higher than the standards prescribed in Western countries, highlighting the need for a thorough review [11].

By mid-2023, with a population exceeding 1.43 billion individuals, India is poised to surpass all other nations and become the world’s most populous country, while also solidifying its position as the third most sought-after manufacturing destination globally [12, 13]. Furthermore, by 2025, it is projected that India will house approximately 25% of the global working population (~ 0.8 billion), a majority of whom, roughly 92%, will be engaged in the informal sector [14–16]. It is within this informal sector that the majority of occupations exposing workers to silica dust are found in India. In 2015-16, an estimated 11.5 million workers in India were employed in occupations associated with silica dust, a number expected to surge to 52 million by the year 2025-26 [14, 17]. Rajasthan’s mining sector is home to roughly 30,000 mines that reaps sandstone, marble, and granite [18]. These mines are spread across several districts of Rajasthan and employ nearly 3 million workers and another 3.2 million are employed in the construction industry [18, 19]. Around 50,000 workers are working in the agate industry in Khambhat, Gujarat [20]. According to recent estimates, the construction industry employs an approximate workforce of nearly 60 million workers in India [21].

Studies have reported varying prevalence rates of silicosis across different regions and occupations among workers exposed to silica dust in India (Table 1) [22–28]. In Haryana, the prevalence was found to be 9% among mine workers,[29] while in Rajasthan, it ranged from 37% among general mine workers to a higher 38–79% among stone mineworkers [18, 24, 25, 30–32]. The introduction of the pneumoconiosis policy in 2019 in Rajasthan led to the diagnosis of 23,436 cases of silicosis out of 192,143 persons screened, indicating a prevalence of 12% among stone carving and stone mining workers, which corresponds to the prevalence reported in sandstone quarries in Rajasthan [11, 19]. The investigators emphasized that their studies in Rajasthan revealed a higher prevalence of silicosis among stone carving workers compared to stone mining workers [6, 19]. In other regions, ordnance factory workers in Delhi had a prevalence of 3.5%,[33] while slate pencil workers in Madhya Pradesh exhibited rates ranging from 25 to 55% [34, 35]. Gujarat, known for its agate and pottery industries, recorded a prevalence varying from 18 to 69% among agate workers and 15% among pottery workers [26, 27, 36]. Silicosis prevalence among stone grinders in Gujarat was reported at 14–18% [37, 38]. Additionally, Mumbai’s flour mill workers had a prevalence rate of 30% [39]. Furthermore, the co-occurrence of silicosis and tuberculosis, referred to as silico-tuberculosis, was observed among mineworkers in Rajasthan at a rate of 7%,[24] while agate workers in Gujarat had a prevalence of 5%, and stone mineworkers as well as stone grinders in Gujarat and Rajasthan recorded rates of 12–25% [25, 37, 38, 40].

**Table 1:** Prevalence of silicosis and silico-tuberculosis in various occupations in India

Disease	Place of Study	Type of Worker	Prevalence	Author-year
Silicosis	Rajasthan	Stone mineworkers	38–79%	Sishodiya 2022 [6], Nandi SS et al. 2021 [25], Sharma DC 2015 [18], Sishodiya et al. 2014 [30], Sishodiya et al. 2014 [31] Sishodiya et al. 2011 [32]
	Gujarat	Agate workers	18–69%	Chaudhury et al. 2010 [26], Rastogi et al. 1991 [36]
	Madhya Pradesh	Slate pencil workers	25–55%	Saiyed et al. 1985 [34], Jain et al. 1977 [35]
	Rajasthan	General mineworkers	37%	Rajavel et al. 2020 [24]
	Mumbai	Flourmill workers	30%	Athavale et al. 2011 [39]
	Gujarat	Stone grinders	14–18%	Tiwari et al. 2010 [38], Tiwari and Sharma 2008 [37]
	Gujarat	Pottery workers	15%	Saiyed et al. 1995 [27]
	Rajasthan	Stone carving and stone mining	12%	Sishodiya 2023 [19]
	Rajasthan	Sandstone quarries	12%	Dhatrak and Nandi 2019 [11]
	Haryana	Mine workers	9%	Govindagoudar et al. 2022 [29]
	Delhi	Ordnance factory workers	3.5%	Viswanathan et al. 1972 [33]
Silico-tuberculosis	Gujarat	Stone grinders	12–25%	Tiwari et al. 2010 [38], Tiwari and Sharma 2008 [37]
	Rajasthan	Stone mineworkers	12%	Nandi et al. 2021 [25]
	Rajasthan	Mineworkers	7%	Rajavel et al. 2020 [24]
	Gujarat	Agate workers	5%	Rupani 2023 [40]

Many challenges exist in the prevention and control of this ancient and preventable disease such as unregulated informal sector, diagnostic difficulties, absence of a surveillance mechanism, inadequate staff, and a lack of awareness [28, 41–44]. Apart from routine dust control measures, several initiatives may be undertaken to control silicosis, including raising awareness, building capacity, improving health systems to treat silicosis patients, integrating silicosis control with tuberculosis control, and strict adherence to statutory regulations [28, 41–44]. Along with the age-old challenges and solutions for silicosis prevention and control, this comprehensive review highlights several newer challenges and solutions.

## Main text

### Challenges

The prevention and control of silicosis in India face significant challenges, including worker-related, disease-related, and systemic challenges (Table 2). One major programmatic challenge is the absence of a comprehensive national health program dedicated to silicosis control, resulting in a lack of coordination among stakeholders and limited surveillance mechanisms.

**Table 2:** Challenges in the prevention and control of silicosis in India

Challenges	Barriers	Description
Worker-related challenges	Lack of awareness	• Lack of awareness about early symptoms • Lack of knowledge about occupational risks
	Lack of PPE use	• Insufficient availability of protective equipment • Inadequate training on PPE usage
	Lack of alternative job	• Limited job opportunities outside silica-dust industries • Lack of skills for alternative employment • Return to dusty work environments
	Lack of access to healthcare	• Geographical barriers to healthcare facilities • Limited healthcare services in remote areas
	Fear of getting laid off	• Job insecurity in case of illness • Fear of losing income and livelihood
Disease-related challenges	Misdiagnosis	• Similar symptoms to other respiratory conditions • Lack of specific diagnostic tests for silicosis
	Alveolar block	• Silica particles obstruct alveoli • Impaired oxygen exchange
	Drugs unable to reach lung tissue	• Fibrosis restricting drug penetration • Reduced effectiveness of medications
	Non-tuberculous mycobacteria	• Co-existence of non-tuberculous mycobacteria • Increased complexity of treatment
	Recurrent pneumothorax	• Frequent lung collapses due to weakened tissue • Higher mortality risk
Systemic challenges	Smaller units	• Non-compliance with regulations in small-scale industries • Limited oversight and enforcement mechanisms
	Unorganized sector	• Lack of structured occupational health and safety practices • Difficulties in implementing regulations • Lack of employer awareness and perception of high costs hinder compliance with periodic silica dust measurements • Absence of public or private agencies offering support for dust measurements • Intention and integrity of regulatory authorities
	Neglect of rehabilitation	• Inadequate support for affected workers’ recovery • Lack of system for relocation to safer environments • Withhold notifications to avoid legal complications • Reluctance to provide compensation to affected employees
	Scarcity of water	• Water shortage affecting dust control measures • Conflicting priorities between water usage and productivity
	Limited knowledge of medical officers	• Insufficient training on occupational health • Inadequate understanding of silicosis diagnosis and management
	Challenges in implementation of pneumoconiosis policy	• Lack of standardized diagnostic protocols • Low awareness and compliance with policy guidelines
	Absence of a national health program	• Underdiagnosis and underreporting • Lack of coordinated efforts among stakeholders • Inadequate surveillance and data collection mechanisms

### Worker-related challenges

#### Job-related issues

People working in the silica-dust industries are mostly contract laborers, making it difficult for them to obtain new employment due to a lack of job opportunities [18, 41, 42, 45–49]. Workers fear that if they are diagnosed with any silica dust-related illnesses, they may be asked to quit, thereby exposing them to unemployment [45, 46]. They do not seek healthcare on time and instead return to dusty work conditions out of fear of getting laid off [42, 44, 46]. Due to the low socioeconomic conditions, vocational rehabilitation of people working in silica dust-related occupations into different occupations is challenging [20]. Moreover, employers do not allow them to take time off to visit the hospital, and workers prefer to consult unqualified doctors in the event of any health-related complaints to avoid wage loss [45, 50].

#### Lack of awareness

Due to a lack of awareness, patients do not suspect a major health problem and believe that silicosis cannot affect them [18, 42, 51–55]. Due to a lack of awareness, coupled with lower education levels that contribute to it, workers in these occupations not only fail to seek appropriate healthcare guidance,[37, 42] but also exhibit a limited inclination towards using personal protective equipment [49, 51–54, 56].

### Disease-related challenges

Silicosis is frequently misdiagnosed as TB [6, 28, 42, 44, 45]. Because the symptoms of tuberculosis (TB) and silicosis are similar, factory workers too believe that they have recurrent TB [18]. As silicosis is incurable, patients’ symptoms continue even after the completion of TB treatment. Upon symptomatic relief, workers return to dusty work settings, increasing their exposure to silica dust even more. The vicious cycle continues, and they experience the progression of silicosis and relapses of TB [40].

Silica dust clogs the alveoli of the lungs,[57] leading to the potentially fatal complication pneumothorax among patients with chronic silicosis [58]. Dormant TB bacilli are occasionally triggered in individuals with silicosis due to macrophage dysfunction and immunological dysregulation, resulting in TB relapses [59, 60]. Because fibrosis of the lung tissue reduces blood flow, TB medications are unable to permeate the tissue and hence reach lower concentrations [59, 61, 62]. Although non-pathogenic, patients with silicosis often demonstrate the presence of non-tuberculous mycobacteria along with the TB bacilli [63–65]. Non-tuberculous mycobacteria are resistant to several of the medications included in the national TB treatment regimen [66].

### Systemic challenges

Control measures aimed at mitigating exposure have generally been implemented and enhanced over time in developed countries like North America and Europe [67]. However, the situation contrasts in developing nations such as China and India, where high levels of exposure persist, leading to a higher prevalence of silicosis [67]. Notable progress has been made in addressing the issue of silicosis in specific occupational settings, such as sandblasting of jeans, with countries like the United States and Germany implementing positive measures like independent factory inspections in response to documented studies [68]. However, it is worth noting that the extent of similar actions being taken in India appears to be limited, highlighting the need for further attention and initiatives in this regard.

The majority of silicosis cases are anticipated to be reported in smaller units and cottage industries [28, 41]. Small enterprises may also be exempt from the statutory norms outlined in the Factories Act, 1948, and the Mines Act, 1952 [20, 28, 41, 44]. As a result, accountability must be assigned among the statutory bodies to execute the provisions of the Factories Act, 1948, the Mines Act, 1952, and the Building and Other Construction Workers (BOCW) Act, 1996, at the smaller units [40]. According to Sect. 112 of the Factories Act, 1948, the Chief Inspector of Factories in each respective state possesses the authority to designate any hazardous unit, regardless of the number of workers employed, as subject to the legal provisions and regulations stated in the Act [69]. As of now, only a limited number of states, such as Gujarat, have taken the initiative to pass resolutions affirming the application of these provisions within their jurisdictions.

Silicosis, recognized as a notifiable and compensable disease under the Factories Act, 1948, and the Mines Act, 1952, remains severely underdiagnosed and underreported in India, as evidenced by the recent annual report of the Ministry of Labour and Employment [69–71]. The report revealed a mere 441 cases of silicosis reported between 2008 and 2022, underscoring the significant extent of underdiagnosis and underreporting within the country [71]. This discrepancy can be attributed to employers’ inclination to withhold notifications from their factories to avoid legal complications and the subsequent obligation to provide compensation to affected employees [20, 41, 44, 72]. There are also challenges surrounding the intention and integrity of the implementing agencies as far as the unorganized sector is concerned [20, 42, 44]. Additionally, there are concerns regarding the outsourcing of medical examinations of workers to doctors who do not understand occupational health [40].

Employers do not allow time for workers to consult a doctor or visit a health facility if they become unwell [42]. Employers also fail to rehabilitate patients and neglect their relocation to less hazardous environments [41, 42]. Concerns have been raised over the scarcity of water for wet drilling in mines and the subsequent decline in drilling efficiency,[44] highlighting the tendency of employers to prioritize the speed of work completion over worker safety. Implementing dust control measures proves costly for industries [44].

In India, the Factories Act of 1948 and the Mines Act of 1952 stipulate the mandatory periodic measurement of dust and silica concentrations in workplaces [69, 70]. However, employers in the country exhibit a lack of awareness regarding these requirements, and the associated measurement processes are perceived as costly. Consequently, many employers in the industrial sector refrain from conducting such measurements and consequently fail to provide relevant data. Furthermore, those employers who are interested in implementing dust measurements often lack knowledge of suitable methods, equipment, and expertise. Additionally, there is a notable absence of public or private agencies that offer support for conducting dust measurements. To address these challenges, the government can play a pivotal role by acting as a facilitator and providing necessary support to enable the implementation of dust measurements in workplaces.

The current state of awareness and clinical suspicion for silicosis among medical officers and clinicians appears to be inadequate, as they exhibit limited knowledge and understanding of several aspects, even after diagnosing a patient with silicosis [44]. Insufficient attention is given to obtaining the occupational history of patients during medical consultations. Consequently, the misdiagnosis and underreporting of silicosis cases are prevalent. The necessity of enhancing the diagnostic skills of doctors in India, specifically in the interpretation of International Labour Organization (ILO) radiographs for the detection of silicosis is emphasized [44].

In India, a standardized diagnostic algorithm and guidelines for silicosis certification are lacking, as is clarification on who may certify a patient as suffering from silicosis [41, 42, 44]. An exception is the western Indian state of Rajasthan, where there is a policy on the detection, prevention, relief, and rehabilitation of pneumoconiosis [6, 73]. However, there are multiple challenges in its implementation, including unawareness, misdiagnosis, and low reporting rates [44]. In the absence of a surveillance mechanism for silicosis, data is not being captured, and the true burden remains elusive [41]. In the absence of a national health program, there is a lack of coordination among various stakeholders involved in silicosis control [44].

### Opportunities

In light of the underreporting of silicosis cases, the actual burden of the disease remains unknown, underscoring the urgent need to enhance diagnostic facilities, provide training to medical officers at primary healthcare centers for accurate diagnosis of silicosis, and establish a robust surveillance mechanism (Table 3). A routine surveillance system would not only generate comprehensive data on the true prevalence of silicosis but also enable monitoring of the disease’s incidence, identification of high-risk areas, and analysis of associated risk factors. By providing timely and reliable information, such a system of surveillance would enable public health authorities to effectively adopt targeted preventive measures and interventions to limit the impact of silicosis.

**Table 3:** Opportunities for prevention and control of silicosis in India

Opportunities	Intervention	Description
Surveillance	Silicosis surveillance	• Establish comprehensive tracking system for silicosis patients • Integrate with TB surveillance • Periodic medical examinations for silicosis and TB
	Active case finding	• Organize diagnostic camps in silica industries • Facilitate early detection of silicosis cases • Conduct surveys to actively search for silicosis cases
	Mapping areas	• Identify and prioritize areas with silica dust industries • Focus control efforts in these areas
Programmatic guidelines	Diagnostic algorithm	• Develop standardized diagnostic algorithms • Ensure accurate and consistent diagnosis with training in ILO radiographs
	Management guidelines	• Establish guidelines for prevention, treatment, and symptom management • Provide treatment options
	Rehabilitation guidelines	• Include guidelines for rehabilitation mechanisms • Address physiotherapy and change of occupation
	Silicosis elimination program	• Establish comprehensive program covering diagnosis, treatment, prevention, awareness, and rehabilitation • Integrate Basic Occupational Health Services (BOHS) with primary health care • Ensure nationwide coverage
	Pneumoconiosis policy	• Expand existing policy to address silicosis nationwide • Emphasize preventive measures, treatment, and support
Preventing silica dust exposure	Alternative uses of silica dust	• Explore alternative applications for silica dust • Reduce overall production and exposure
	Silica dust disposal	• Establish proper disposal systems for wet dust • Deep burial
	Subsidy for silica dust disposal	• Provide subsidies for dust disposal systems • Encourage compliance
	Exhaust system	• Develop efficient exhaust systems • Capture and remove silica dust
	Water spraying system	• Promote use of water spraying systems • Reduce silica dust in workplaces
	Multi-sectoral coordination	• Coordinate health, labor, and environmental departments • Tackle silicosis collectively
	Dedicated place for all workers	• Create industrial estates near cottage industries • Facilitate easy registration and medical examinations
	Personal protective equipment (PPE)	• Promote use of masks, goggles, and gloves
	Dust reduction at source	• Implement control measures at the source • Follow hierarchy of control measures • Integrate AI-ML technologies for effective dust reduction
	Alternative job	• Facilitate shift to alternative jobs • Eliminate dust exposure
	Substitution of hazardous material	• Encourage use of less hazardous materials
	Enclosed workspaces	• Enclose areas generating silica dust • Limit exposed workers
	Widespread prevention activities	• Conduct extensive awareness and prevention activities • Target workers in industries
Effective law enforcement	Law enforcement for smaller units	• Increase personnel in the Ministry of Labour and Employment (Government of India) • Strengthen enforcement in smaller units • Enhance monitoring of compliance with safety measures • Focus on high-risk areas
	Ethical law enforcement	• Address concerns regarding intention and integrity of implementing agencies • Focus on unorganized sector
	Qualified personnel for law enforcement	• Ensure presence of qualified medical personnel • Expertise in occupational health • Short course on Occupational Health in MBBS curriculum

### Surveillance

Identifying and mapping locations in India where industries generating silica dust are situated would be the first step toward eliminating the disease [22, 41]. To facilitate effective monitoring, it is essential to define priority districts and sub-district areas/clusters [22, 41]. Considering that a significant portion of these factories operates in the informal sector, tracking efforts would help concentrate control activities specifically on these areas, ensuring targeted implementation of preventive and control measures.

A surveillance system needs to be established for the early detection and tracking of all patients with silicosis [2, 6, 28, 74, 75]. Organizing diagnostic camps in industries where silica dust is generated would also aid in the early detection of silicosis. It is suggested that active case finding, which is currently a part of the national TB program, be expanded to include silicosis [40, 44]. It is a statutory requirement to conduct periodic medical examinations of workers exposed to silica dust every six months [6, 22, 27, 69, 70, 75, 76]. Considering the high prevalence of TB among silica dust-exposed individuals, TB surveillance should be a part of periodic medical examinations [27].

### Preventing silica dust exposure

#### Silica dust reduction

Several studies conducted in India have consistently reported silica dust levels exceeding the prescribed limits set by various agencies, ranging from 0.025 to 0.15 mg/m^3^ [11, 77]. Additionally, lower but still significant levels have been identified, emphasizing the importance of minimizing occupational exposure to mitigate the risk of silicosis [78]. Within specified mining sectors, such as limestone (0.015 mg/m^3^), iron (0.012 mg/m^3^), and bauxite (0.008 mg/m^3^) mines, crystalline silica concentrations were found to be below the prescribed permissible exposure limit (PEL) of 0.15 mg/m^3^ for Indian mines [78]. Conversely, in sandstone mines, concentrations were slightly above the PEL (0.17 mg/m^3^) and in granite and masonry mines, they were lower than the PEL (0.12 mg/m^3^) [77]. Nevertheless, researchers suggest the need for a lower PEL in India, as even at mean levels of 0.12 mg/m^3^, cases of silicosis were reported among workers [11]. Notably, stone crushing sites (0.098–2.29 mg/m3), coal mines (2.7–5.3 mg/m3), and agate workers (0.11–0.12 mg/m3) exhibited silica dust concentrations well above the limits established by ACGIH and OSHA, which range from 0.025 to 0.05 mg/m^3^ [36, 79–82]. Moreover, other studies documented significantly high levels of silica dust concentrations among slate pencil workers (55–57%), quartz manufacturers (26%), and stone crushers (19%) [34, 83, 84]. Among stone mine workers with TB, silica dust concentrations ranged between 0.11 and 0.16 mg/m^3^, further emphasizing the critical need to reduce occupational exposure to silica dust in order to mitigate the prevalence of TB [85].

Currently, various preventive measures are in place to reduce silica dust, including the use of ventilation systems, water suppression techniques, dust control equipment, and personal protective equipment such as masks. Exhaust systems should be implemented in factories where silica dust is generated [2, 22, 48, 55, 75, 86–89]. Dust control measures have been recognized as an effective engineering control measure for reducing worker exposure to silica dust [48, 86, 88]. Installing exhaust fans in factories where silica dust is generated might also be beneficial [2]. It is critical to make such systems cost-effective, as high operational costs are a deterrent to using such exhaust systems [43, 44, 86, 90, 91]. Many factories/cottage industries use a water spraying technique, often known as a wet process [2, 20, 22, 42, 82, 87–89, 92, 93]. A constant flow of water droplets is placed over the spot where the stones are polished. Alternatively, in mines, some drilling equipment only operates when water is supplied to them. Places, where the silica dust is generated in factories, should be enclosed so that only a small number of workers are exposed to it. In addition, only a restricted number of workers should be permitted in sections of the factory where dust is formed. Technological advances such as artificial intelligence and machine learning might help industries implement effective and cost-efficient dust control measures [94, 95]. While technological solutions for dust control are readily available, the lack of enforcement mechanisms and the attitude of both employers and employees towards implementing these measures pose significant obstacles to their effective adoption.

#### Silica dust alternatives

Alternative uses for silica dust generated in the industry should be investigated. The generated dust can be utilized as fillers in the manufacturing of concrete and construction materials [96]. Because of the water spraying mechanism, silica dust is settling on the ground as wet dust. However, as the wet dust dries, it becomes dry silica dust. As a result, wet dust should be disposed of using a deep burial technique. Employers should be offered subsidies to help them set up silica dust disposal systems. Alternatively, a more hazardous material (for example, stones) should be substituted with a less hazardous material.

#### Prevention activities

In the absence of effective treatments, widespread preventative activities are recommended for silicosis [75]. A part of the total budget allocated by the government for rehabilitative activities should be targeted for preventive efforts. An industrial estate should be created near the houses of employees in cottage industries so that all workers may work together [48, 97]. Because all workers would be in one location, the procedure of registering workers and undertaking preventative activities and periodic medical checks would be streamlined [48]. The state government of Gujarat had planned to relocate cottage industries of agate workers from Khambhat city to an alternative location, offering land and necessary infrastructure [48]. Additionally, a cooperative society was proposed to address environmental and occupational health concerns, with the owners taking responsibility [48]. However, the implementation of this plan has been delayed due to potential challenges related to land allotment and monitoring.

Integrating Basic Occupational Health Services (BOHS) with primary health care can provide an effective approach to the prevention and control of silicosis [98]. The integration of BOHS with primary health care can ensure that workers are regularly screened for early detection of silicosis, and appropriate treatment is provided [98]. This can also ensure that the community is aware of the health risks associated with exposure to silica dust and the preventive measures to reduce exposure [98]. Additionally, to tackle the problem of silicosis, coordination is essential across multiple stakeholders such as health, labor and employment, and pollution control boards [22, 42, 76]. In India, the district collector (administrative head of a district) should promote such collaboration among several departments for silicosis prevention and control.

#### Employer’s role

To prevent any future dust exposure, the workers must be moved to alternative job options within the same factory that offers reduced or no exposure to silica dust, considering factors such as factory layout, job-specific hazards, and other relevant factors that could influence exposure levels. Implementing job-shifting practices can present practical challenges that need to be carefully addressed. Government regulatory bodies, on the other hand, must guarantee that factory owners relocate workers to another vocation as soon as they exhibit signs of silicosis. It is critical not to dismiss the individual from his employment, since this would result in further malnutrition and the spread of other diseases. As a result, such a work transition should ensure that their source of income stays intact.

Reduction of dust at the source through elimination, substitution, engineering control, administrative control, and lastly the use of personal protective equipment is recommended [2, 74, 88, 99, 100]. It is reiterated that this should be the factory’s hierarchy of control measures employed for dust control [100]. Industrial hygiene procedures are critical for reducing silica dust at the source and in its journey in the air before it reaches the worker. Personal protection equipment such as masks, goggles, hand gloves, and respirators are recommended for decreasing worker exposure to silica dust [2, 22, 88, 99].

### Effective law enforcement

It is vital to post qualified personnel in implementing agencies to execute the provisions of various legislative Acts [42]. The certifying surgeons assigned to the implementing agencies are merely MBBS doctors with limited understanding, expertise, or credentials in occupational health [42, 44]. Courses such as the Associate Fellow in Industrial Health (AFIH), offered by several institutes under the supervision of the Ministry of Labor and Employment, educate medical officers on various aspects of occupational health. Larger portions of occupational health must be incorporated into the MBBS curriculum so that medical officers have an understanding before beginning their profession or being recruited [22, 28, 41, 42, 74–76]. A short course in integrity and ethics might improve their service delivery at work. Furthermore, it is imperative to augment the workforce in the relevant departments of the Ministry of Labour and Employment (Government of India) to ensure an adequate number of personnel available for conducting regular visits to industries and effectively monitoring compliance with safety measures [20].

### Programmatic guidelines

Standardized guidelines on diagnosis, symptomatic therapy, comorbidity management, and palliative care should be developed and circulated widely. Rehabilitation mechanisms such as physiotherapy, oxygen therapy, and other alternative therapies should be a part of the standardized document. Considering that health is under the purview of individual states in India, it is advisable for other states, following the example of Rajasthan, to consider implementing a monetary compensation policy for individuals affected by silicosis [6, 28, 73]. The pneumoconiosis policy in Rajasthan was established after a decade of concerted efforts and representations by various government organizations, including the Rajasthan State Human Rights Commission and the Association for Rural Advancement through Voluntary Action and Local Involvement (ARAVALI, https://aravali.org.in/publicationsresearch.html), as well as non-governmental organizations like the Mine Labour Protection Campaign (https://minelabour.org/) [6, 22, 73, 101]. These entities brought attention to the high prevalence of silicosis among stone mine workers in the state and emphasized the necessity for financial rehabilitation. Factors such as a higher prevalence of silicosis cases, increased awareness of the issue, and proactive efforts by local authorities contributed to the adoption of these policies. While the prevalence of silicosis may vary across different states in India, the decision to implement proactive measures depends on factors such as political will and resource availability. Therefore, it is essential to encourage other states to follow Rajasthan’s example and take decisive steps towards mitigating the burden of silicosis in their respective regions.

A pneumoconiosis elimination program encompassing health promotion, personal protection, diagnostic criteria, preventive measures, symptomatic management, prevention of silica dust exposure, treatment, and rehabilitation is felt important for wider adoption [28]. A distinct ‘National Silicosis Control Program’ with reporting and review/monitoring, would result in program activities being funded under a separate budget head [25, 28, 41]. Until such a program is established, silicosis control activities should be integrated with TB elimination efforts [22, 28, 40, 48, 76].

## Conclusions

Silicosis continues to be a fatal occupational disease, with serious consequences on workers’ productivity and quality of life. Silicosis is not a state-specific issue in India; it is a national one. Silica dust exposure and its consequences are fully preventable, with the benefits of prevention considerably outweighing the benefits of treating patients with silicosis. Data from periodic medical examinations of workers in silica-dust-producing industries should be analyzed to generate robust evidence that can support and direct targeted interventions aimed at mitigating the risks associated with silica dust exposure. Not only should employees and employers be educated about the fatal disease, but also doctors, including interns and rural medical officers, need to be well-informed. Preventing worker exposure to silica dust will have significant financial implications (e.g., through improved worker productivity, reduced healthcare costs, and minimized loss of workdays) and provide an impetus to India’s untapped potential - a healthy labor force. With India being the third-most sought-after manufacturing destination globally, a comprehensive national health program on silicosis would be a necessary intervention to protect workers’ health.

## Data Availability

There was no primary or secondary data collected for this review article.

## List of abbreviations

AFIH: Associate Fellow in Industrial Health
BOCW: Building and Other Construction Workers
DALY: Disability Adjusted Life Years
MBBS: Bachelor in Medicine and Bachelor in Surgery
TB: Tuberculosis
